# Preoperative Folate Receptor-Positive Circulating Tumor Cell Level Is a Prognostic Factor of Long Term Outcome in Non-Small Cell Lung Cancer Patients

**DOI:** 10.3389/fonc.2020.621435

**Published:** 2021-01-28

**Authors:** Hang Li, Bin Li, Yunjian Pan, Yang Zhang, Jiaqing Xiang, Yawei Zhang, Yihua Sun, Xiang Yu, Wei He, Hong Hu

**Affiliations:** ^1^Department of Thoracic Surgery and State Key Laboratory of Genetic Engineering, Fudan University Shanghai Cancer Center, Shanghai, China; ^2^Institute of Thoracic Oncology, Fudan University, Shanghai, China; ^3^Department of Oncology, Shanghai Medical College, Fudan University, Shanghai, China; ^4^Department of Medicine, Geno Biotech Co. Ltd., Shanghai, China

**Keywords:** circulating tumor cell, folate receptor, non-small cell lung cancer, surgery, prognosis

## Abstract

**Background:**

Surgical resection is often the preferred treatment for non-small cell lung cancer (NSCLC) patients. Predictive biomarkers after surgery can help monitoring and treating patients promptly, so as to improve the clinical outcome. In this study, we evaluated one potential candidate biomarker, the folate receptor-positive circulating tumor cell (FR^+^CTC), by investigating its prognostic and predictive significance in NSCLC patients who underwent surgery.

**Methods:**

In this prospective, observational study, we enrolled NSCLC patients who were eligible to receive surgery. Prior to operation, peripheral blood was collected from each patient for an FR^+^CTC analysis. FR^+^CTCs were isolated by negative enrichment using immunomagnetic beads to deplete leukocytes and then quantitatively detected by a ligand-targeted polymerase chain reaction (PCR) method. These patients were then given standard care and were actively followed up for seven years. At the end of the follow-up period, the association between the FR^+^CTC level and the prognosis in these patients was evaluated.

**Results:**

Overall, preoperative FR^+^CTC level was not significantly different among NSCLC patients with adenocarcinoma or non-adenocarcinoma subtypes (*P* = 0.24). However, between patients with low- and high-risk pathological adenocarcinoma subtypes, the preoperative FR^+^CTC level was significantly different (*P* = 0.028). Further, patients with lower preoperative FR^+^CTC level had longer relapse-free survival (RFS) and overall survival (OS) than those with higher preoperative FR^+^CTC level (RFS: not reached vs. 33.3 months, *P* = 0.018; OS: not reached vs. 72.0 months, *P* = 0.13). In a multivariate COX regression analysis, FR^+^CTC level (HR = 4.10; 95% CI, 1.23–13.64; *P*=0.022) and pathological stage (HR = 3.16; 95% CI, 1.79–10.14; *P* = 0.0011) were independent prognostic factors of RFS. Moreover, FR^+^CTC level together with adenocarcinoma subtypes provided additional information on risk for disease recurrence compared with FR^+^CTC or adenocarcinoma subtype alone.

**Conclusion:**

Our study demonstrated that the preoperative FR^+^CTC level was a potential predictor for the prognosis of NSCLC patients underwent surgery. Further, when preoperative FR^+^CTC level is considered together with primary tumor proliferation characteristics, its prognostic value supplements that of these conventional pathological features.

## Introduction

Non-small cell lung cancer (NSCLC) is a leading cause of cancer-related death worldwide (1, 2). Surgical resection is usually the preferred treatment option for NSCLC patients who are eligible. In general, for patients with stages 0-I NSCLC, five-year survival rate can be as high as 90% (3). The five-year survival rate drops to 60 and 40% for stage II and III NSCLC, respectively (3). In fact, for patients with metastasis and recurrence, the 5-year survival rate is only 15% (3). However, even for early stage NSCLC patients with R0 resection, their prognosis may vary significantly. As such, identifying patients who are at risk of developing recurrence after surgery can help better manage these patients postoperatively and improve their clinical outcomes. However, few sensitive biomarkers to predict early recurrence or metastasis postoperatively are available, limiting the options and timeliness of treating high-risk patients during their follow-up.

The prospects of circulating tumor cells (CTCs) as a “liquid biopsy” diagnostic tool are attractive given the difficulties in obtaining adequate tissue for pathological analysis in selected individuals. Over the last decade, as more sensitive and reliable methods for CTC detection were developed and adopted in practice, the clinical utilities of CTC have been established and well accepted by practitioners worldwide (4, 5). For instance, CTC has proven a significant prognostic factor in metastatic breast cancer, colorectal cancer, prostate cancer, NSCLC, and a few other cancer types (6–9). In lung cancer, the use of CTC in guiding clinical decision making is frequent and productive. A number of studies have demonstrated that CTCs can be detected at all stages of lung cancer, and in certain instances even prior to the definitive identification of the primary cancer (10, 11). In other studies, CTCs have been shown to predict treatment responses and prognosis in NSCLC (11–13).

Because it is highly expressed in a number of solid tumor types, folate receptor (FR) has been extensively studied as a drug target (14). It has also been pursued as a biomarker for *in vivo* imaging of ovarian cancer and the development of CTC detection method (15). Previous studies showed that folate receptor-positive CTCs (FR^+^CTCs) have both high sensitivity (72–78%) and specificity (82–90%) for the diagnosis of lung cancer (16, 17). Further, in patients with advanced, EGFR-positive NSCLC, high level of FR^+^CTC (≥ 17 FU/3 ml) prior to first-line EGFR tyrosine kinase inhibitor treatment was associated with poorer prognosis compared to lower baseline level of FR^+^CTC (< 17 FU/3 ml) (18). Similarly, in small cell lung cancer patients receiving first-line chemotherapy, those having a higher baseline FR^+^CTC level prior to treatment initiation had significantly shorter progression free survival (19). In a study on early stage NSCLC patients underwent surgery, Zhou et al. demonstrated that preoperative FR^+^CTC level was associated with tumor invasion and, when combined with maximum tumor diameter, could satisfactorily distinguish adenocarcinoma *in situ* (AIS) from minimally invasive adenocarcinoma (MIA) and invasive pulmonary adenocarcinoma (20).

In the present study, we evaluated preoperative FR^+^CTC level in peripheral blood from NSCLC patients through the ligand-targeted (LT) PCR method as described previously (10, 11). A 7-year follow-up was conducted to assess the association between preoperative FR^+^CTC level and long-term survival.

## Materials and Methods

### Study Subjects

This is a single-center, prospective, observational study designed to assess the long-term prognostic value of FR^+^CTC level in NSCLC patients. A total of 62 patients with NSCLC who were scheduled to receive surgical resection in Fudan University Shanghai Cancer Center were enrolled from May 2012 to August 2012. All patients received FR^+^CTC analysis preoperatively. Inclusion criteria were as follows: 1) the patient was between 18 and 80 years old; 2) the final pathological diagnosis confirmed NSCLC; 3) the patient’s Eastern Cooperative Oncology Group score was 0–2; 4) the initial surgical treatment was R0 resection; and 5) sufficient amount of peripheral blood sample was collected from the patient for FR^+^CTC analysis within 1 day before surgery. Exclusion criteria were as follows: 1) the patient had other malignant tumors in the past; 2) the patient received adjuvant chemotherapy. Patients were regularly followed up for seven years after surgery and were provided with standard care during the follow-up period. Two patients who had other malignant tumors in the past and six patients who had received adjuvant chemotherapy were excluded. In total, 54 patients were included in the prognosis analysis. The study was approved by the ethics committee of Shanghai Cancer Center Fudan University (050432-4-1911D) and written informed consent was obtained from all patients.

### FR^+^CTC Analysis

CTCs were analyzed and quantified by the use of the CytoploRare Kit (Genosaber Biotech, Shanghai, China). Three ml of peripheral blood were withdrawn into an EDTA-containing anti-coagulant tube from each subject one day before surgery. CTCs were enriched by lysis of erythrocytes followed by immuno-magnetic depletion of leukocytes from the whole blood. Then, FR^+^CTCs in each sample were quantified by ligand-targeted polymerase chain reaction (LT-PCR) as previously described (11, 17, 21). The primer sequences were as follows: detection probe (an oligonucleotide that is conjugated to the tumor-specific ligand folic acid), 5’–CTCAA CTGGT GTCGT GGAGT CGGCA ATTCA GTTGA GGGTT CTAA–3’; forward primer, 5’–TATGA TTATG AGGCA TGA–3’; reverse primer, 5’–GGTGT CGTGG AGTCG–3’; TaqMan probe, 5’–FAM–CAGTT GAGGG TTC–MGB–3’. The LT-PCR reaction was performed on an ABI 7500 Real-time PCR under the following conditions: denaturation at 95 °C for 2 min, annealing at 40 °C for 30 s, extension at 72 °C for 30 s, and then cooling at 8 °C for 5 min; 40 cycles of denaturation at 95 °C for 10 s, annealing at 35 °C for 30 s, and extension at 72 °C for 10 s. A self-referenced CTC unit (denoted “FU”) derived from standard curve was used to indicate the abundance of FR^+^CTCs in 3 ml peripheral blood. For examples, 8.7 FU indicates 8.7 FU in 3 ml of whole blood. A serial of standards containing oligonucleotides (10^−14^ to 10^−9^ M, corresponding to 2 to 2×10^5^ CTC units/3 ml blood) are used for FR^+^CTC quantification.

### Follow-Up

Postoperative treatment and follow-up were carried out according to the NCCN Guidelines for NSCLC. Patients’ demographics, tumor characteristics, surgical information, and survival outcomes were collected in the medical record system. Imaging evaluation of recurrence or metastasis was performed for all patients during their follow-up according to the Response Evaluation Criteria in Solid Tumors (RECIST V 1.1, 2009) (22). Specifically, for Stage II and III patients, contrast chest CT and ultrasound of neck and abdomen were performed every 3 months. For Stage I patients, these examinations were performed every 6 months. All patients received brain MRI every 12 months. Telephone follow-up was conducted if an in-clinic follow-up visit was not feasible. Follow-up was conducted with each patient until death or September 2019. Relapse free survival (RFS) was defined as the period between the time of surgery and cancer recurrence or metastasis. Overall survival (OS) was defined as the period between the time of surgery and death from any cause.

### Statistical Analysis

Clinicopathological characteristics including age, sex, tumor size, pathological type, tumor differentiation, adenocarcinoma subtype, and TNM stages were collected. According to the degree of invasion and adenocarcinoma subtype, patients were divided into two groups (Low Risk Group: AIS, MIA, lepidic, and acinar; High Risk Group: mucinous, micropapillary, and solid) (23, 24).

Categorical data were presented as counts and percentages and compared using Fisher’s exact test. FR^+^CTC levels were presented as medians with interquartile ranges and compared using Kruskal-Wallis test or Mann-Whitney U test. The most efficient cutoff values of FR+CTC level to stratify the study population into different prognostic groups were identified using maximally selected rank statistics (R package “maxstat” https://cran.r-project.org) (25). Survival curves were estimated by Kaplan-Meier method and compared using the log-rank test. Risk factors potentially affecting the survival were assessed by Cox proportional hazard regression analysis. Potentially significant covariates (*P* < 0.2) in the univariate analysis were selected for subsequent multivariate analysis, where a backward stepwise method was applied to investigate the effect of FR^+^CTC level on survival.

All statistical analyses were performed using R 4.0.0 (R Foundation for Statistical Computing, Vienna, Austria, https://cran.r-project.org). A *P* value less than 0.05 was considered to be statistically significant.

## Results

The study included 54 NSCLC patients who underwent surgery between May and August 2012 and were regularly followed up postoperatively for up to 7 years (**Table 1**). The average (± standard deviation, SD) age of the patients was 60.6 ± 10.2 years old. The average tumor size of these patients was 3.1 ± 2.0 cm. Pathological examinations indicated that 9.3% of the patients had pre-invasive lesion, 61.1% had adenocarcinoma, 20.4% had squamous cell carcinoma, and 9.2% had large cell carcinoma or adenosquamous carcinoma (**Table 1**). There was no significant difference in the patients’ age, gender, tumor size, pathological type (AIS/MIA, adenocarcinoma, squamous cell carcinoma, large cell carcinoma, and adenosquamous carcinoma), tumor differentiation, T stage, N stage, or pathological TNM stage between two FR^+^CTC (High v. Low, as defined in Section 3.1) groups (**Table 1**).

**Table 1:** Clinicopathological Characteristics of 54 NSCLC Patients.

Characteristics	Patients n = 54, No. (%)	FR ^+^CTC≤7.9 FU/3 ml n = 14, No. (%)	FR ^+^CTC>7.9 FU/3 ml n = 40, No. (%)	*P* value
**Age** (years-old, mean ± SD)	60.6 ± 10.2	60.4 ± 11.2	60.7 ± 10.0	>0.999
<60	21 (38.9%)	5 (35.7%)	16 (40.0%)	
≥60	33 (61.1%)	9 (64.3%)	24 (60.0%)	
**Sex**				0.7725
Male	30 (55.6%)	8 (57.1%)	22 (55.0%)	
Female	24 (44.4%)	6 (42.9%)	18 (45.0%)	
**Tumor size** (cm, mean ± SD)	3.1 ± 2.0			0.7812
<3 cm	27 (50%)	9 (64.3%)	18 (45.0%)	
≥3 cm	27 (50%)	5 (35.7%)	22 (55.0%)	
**Pathological type**				NA
AIS/MIA	5 (9.3%)	1 (7.1%)	4 (10%)	
invasive adenocarcinoma	33 (61.1%)	9 (64.3%)	24 (60.0%)	
squamous cell carcinoma	11 (20.4%)	2 (14.3%)	9 (22.5%)	
large cell carcinoma	3 (5.5%)	2 (14.3%)	1 (2.5%)	
adenosquamous carcinoma	2 (3.7%)	0	2 (5%)	
**Tumor differentiation^a^**				0.4330
High	7 (14.6%)	3 (25.0%)	4 (11.1%)	
Middle/Middle-Low	26 (54.2%)	5 (41.7%)	21 (58.3%)	
Low	15 (31.2%)	4 (33.3%)	11 (30.6%)	
**Adenocarcinoma subtyp^a^**				0.2374
Leptic/Acinar	18 (%)	7 (77.8%)	11 (50.0%)	
Mucinous/micropapillary/Solid	13 (%)	2 (22.2%)	11 (50.0%)	
**T stage^a^**				>0.9999
Tis+T1	26 (55.3%)	7 (58.3%)	19 (54.3%)	
T2+T3	21 (44.7%)	5 (41.7%)	16 (45.7%)	
**N stage^a^**				0.7337
**N0**	31 (59.6%)	7 (58.3%)	14 (46.7%)	
N1-2	21 (40.4%)	5 (41.7%)	16 (53.3%)	
**Pathological TNM stage**				>0.9999
0+I	26 (48.1%)	7 (50.0%)	19 (47.5%)	
II+III	26 (48.2%)	6 (42.9%)	20 (50.0%)	
IV	2 (3.7%)	1 (7.1%)	1 (2.5%)	

^a^Not all patients have this information.

NA, not applicable; AIS, adenocarcinoma in situ; MIA, minimally invasive adenocarcinoma.

By the last follow-up visit, 27 (50%) patients had developed recurrence and 21 (38.9%) patients had died. The median RFS was 55.2 months and the median OS was not reached. The five-year recurrence free survival and overall survival were 49.3 and 64.2%, respectively.

### Prognostic Significance of FR^+^CTC Levels

**Figure 1:**
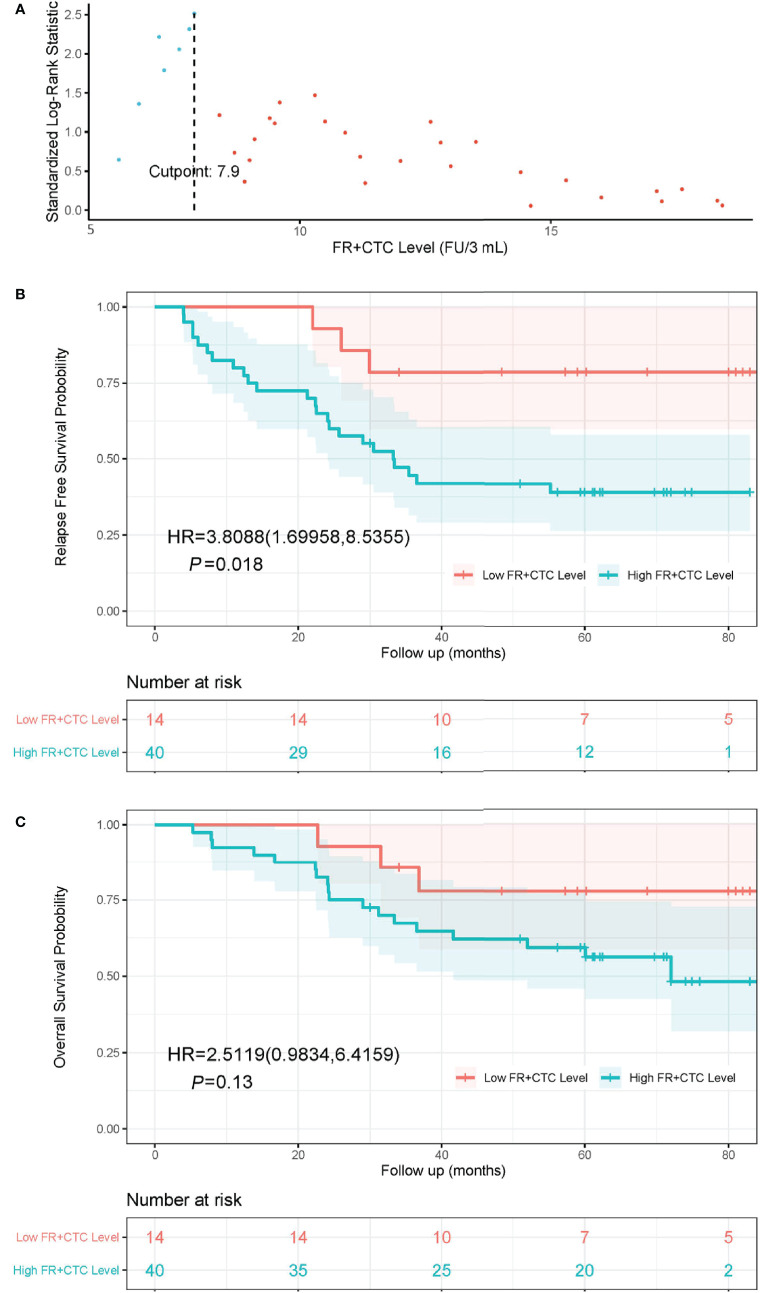
The cutoff value of FR^+^CTC level on prognosis and Kaplan-Meier curves of RFS and OS in NSCLC patients with different preoperative FR^+^CTC level. **(A)** The standardized statistic of integer valued log-rank scores as a function of the hypothetical cutpoint of FR^+^CTC level. The process obtains its maximum of 2.5 at 7.9 FU/3 ml as indicated by the dashed line. **(B)** RFS curve in patients with low (≤ 7.9 FU/3 ml) and high (> 7.9 FU/3 ml) FR^+^CTC level. **(C)** OS curve in patients with low (≤ 7.9 FU/3 ml) and high (> 7.9 FU/3 ml) FR^+^CTC level.

The optimal cutoff FR+CTC level was determined using the maximally selected rank statistics (MSRS). At the cutoff of 7.9 FU/3 ml blood, the MSLS reached its local maximum at 2.5 (**Figure 1A**, indicated by the dashed line). Hence, we chose 7.9 FU/3 ml as the optimal cutoff and separated patients into the High FR^+^CTC Group (≥ 7.9 FU/3 ml) and the Low FR^+^CTC Group (< 7.9 FU/3 ml).

The Kaplan–Meier curves of the High FR^+^CTC Group and the Low FR^+^CTC Group were provided in **Figures 1B** and **1C**. The median RFS was 33.3 months in the High FR^+^CTC Group and not reached in the Low FR^+^CTC Group, respectively (HR = 3.81; 95% CI 1.70 to 8.54, *P* = 0.018) (**Figure 1B**). The median OS was 72.0 months in the High FR^+^CTC and not reached in the Low FR^+^CTC Group (HR, 2.51; 95% CI, 0.98 to 6.42; *P* = 0.13) (**Figure 1C**).

### FR^+^CTC Levels and Pathological Subtypes

**Figure 2:**
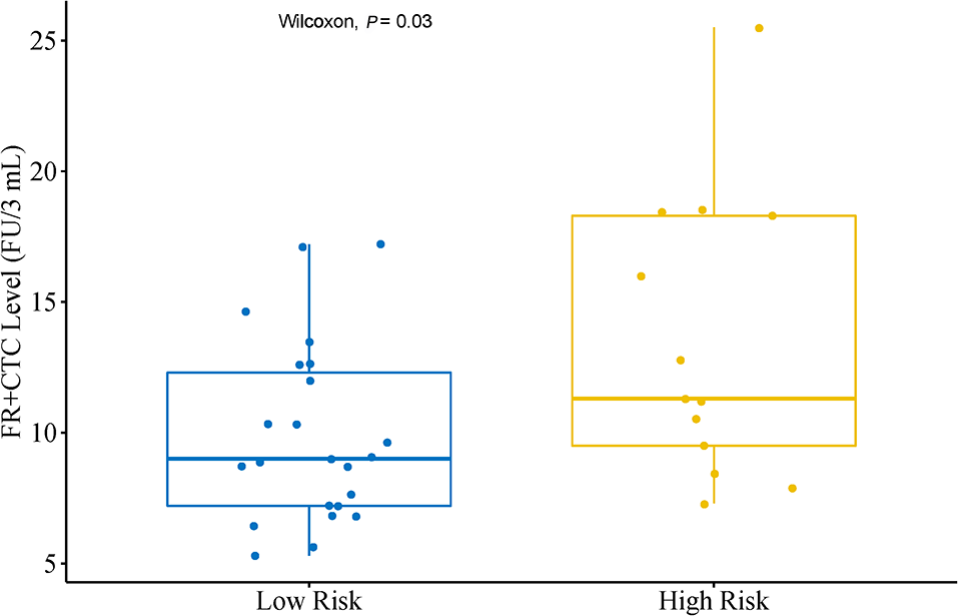
Distribution of FR^+^CTC level in lung adenocarcinoma patients with different subtypes. Low Risk Group (n = 23) including patients with adenocarcinoma in situ, minimally invasive adenocarcinoma and invasive adenocarcinoma predominantly showing lepidic or acinar growth pattern. High Risk Group (n = 13) including patients with invasive mucinous adenocarcinoma and invasive adenocarcinoma predominantly showing micropapillary or solid growth pattern.

In a subgroup analysis, we first divided patients into an Adenocarcinoma Group and an Other Group. The former included pathological subtypes adenocarcinoma, while the latter included pathological subtypes squamous cell carcinoma, large cell carcinoma, and adenosquamous carcinoma. There was no significant difference in FR^+^CTC level between these two subgroups (*P* = 0.24). Patients in the Adenocarcinoma Group were further grouped into a High Risk Group and a Low Risk Group based on their pathological subtypes. The High Risk Group included invasive mucinous adenocarcinoma, and invasive adenocarcinoma predominantly showing micropapillary or solid growth pattern. The Low Risk Group included AIS, MIA, and invasive adenocarcinoma predominantly showing lepidic or acinar growth pattern. **Figure 2** showed the FR^+^CTC levels in the two risk groups. The High Risk Group had significantly higher FR^+^CTC level than the Low Risk Group [median = 11.3 FU/3 ml, interquartile range (9.0, 18.4) vs. median = 9.0 FU/3 ml, interquartile range (7.2, 12.6), *P* = 0.028].

### Cox Regression Analysis

**Figure 3:**
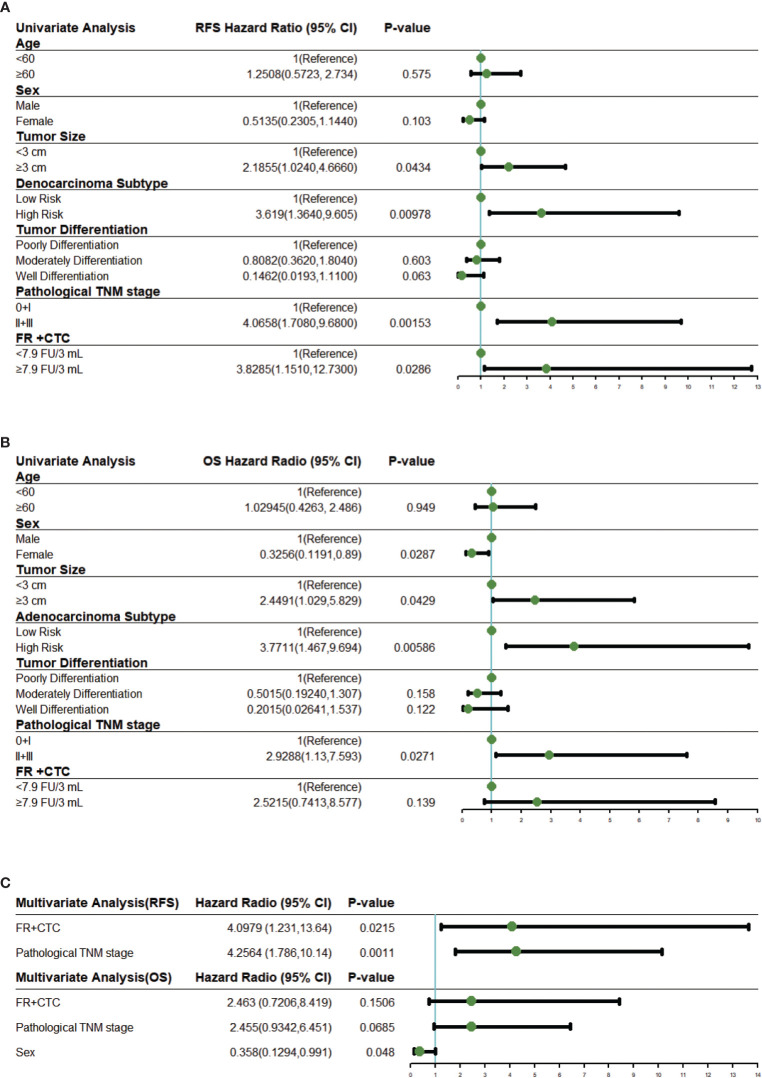
Univariate and multivariate Cox regression analysis. **(A)** Forest plots showing the results of univariate regression analysis for RFS. **(B)** Forest plots showing the results of univariate regression analysis for OS. **(C)** Forest plots showing the results of multivariate regression analysis for RFS and OS. The x axis represents the hazard radio, and the reference line (blue) and significance were calculated using a Cox proportional hazards model. Error bars, 95% CIs. CI, confidential interval.

Univariate COX proportional hazard regression analysis for RFS suggested that survival rates differed between sex, tumor size, adenocarcinoma subtype, tumor differentiation, pathological TNM stage, and FR^+^CTC level (**Figure 3A**). These variables were further included in multivariate COX regression analysis. Adenocarcinoma subtypes favorably affected RFS in the univariable model but was not enrolled into the multivariable model due to limited number of patients. As shown in **Figure 3C**, a multivariate COX proportional hazard regression model demonstrated that high FR^+^CTC level (HR, 4.10; 95% CI, 1.23 to 13.64; *P* = 0.022) and stage II/III disease (HR, 4.26; 95% CI, 1.79 to 10.14; *P* = 0.0011) were associated with shorter RFS.

Similar analyses were performed on these variables for the OS. In the univariate analysis, the FR^+^CTC level was also associated with the OS (**Figure 3B**). The multivariate regression analysis revealed that gender (HR, 0.36; 95% CI, 0.13 to 0.99, *P* = 0.048) was the only independent prognostic factor for the OS (**Figure 3C**). Patients with low FR^+^CTC level or pathological TNM stage were associated with longer OS, although such an association did not reach statistical significance (*P* = 0.15 and 0.067, respectively, **Figure 3C**).

### Prognostic Significance of Adenocarcinoma Subtypes

**Figure 4:**
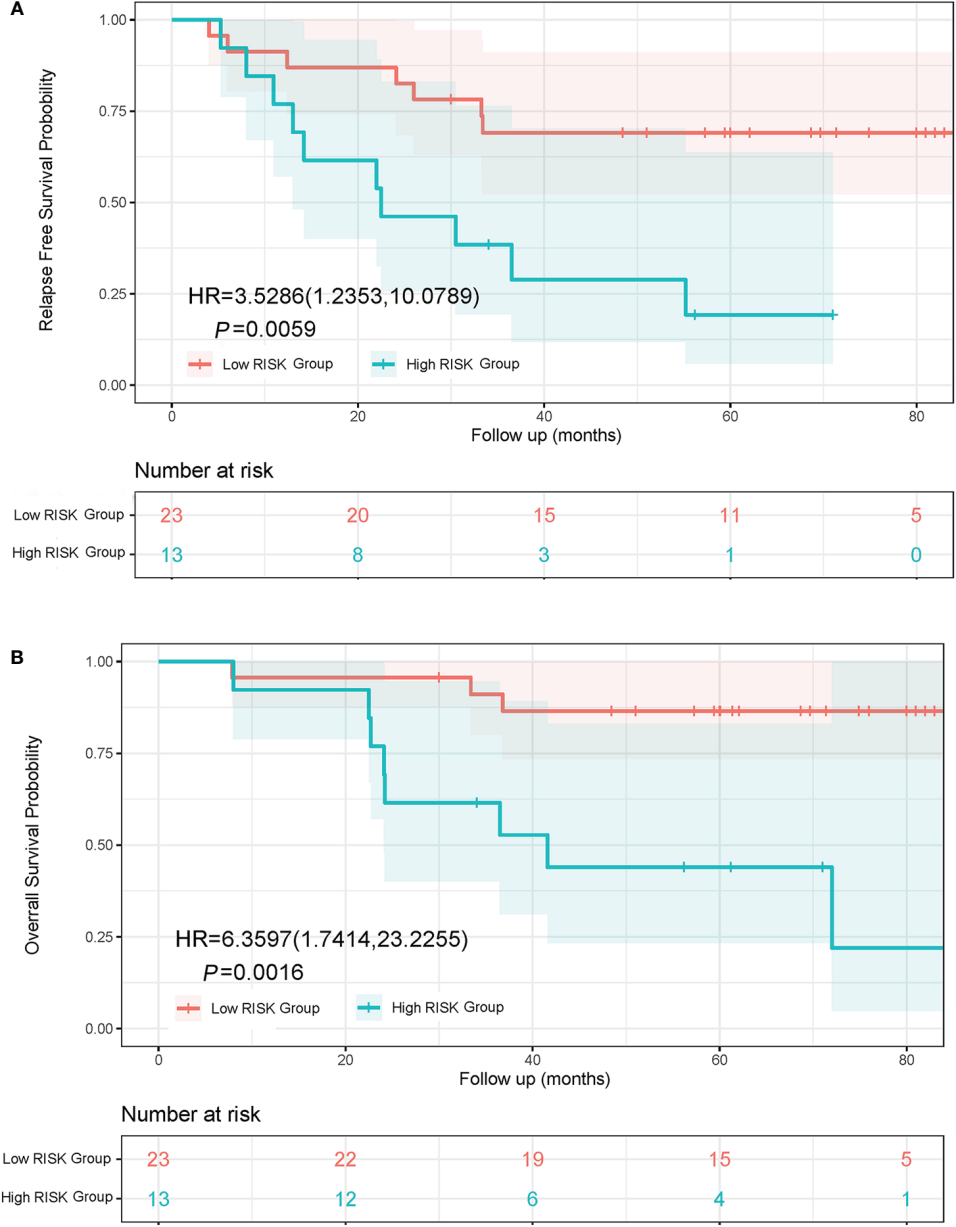
Kaplan-Meier curves of RFS and OS in lung adenocarcinoma patients with different subtypes. **(A)** RFS curve of Low Risk Group and High Risk Group. **(B)** OS curve of Low Risk Group and High Risk Group. The Low Risk Group included adenocarcinoma in situ, minimally invasive adenocarcinoma, and invasive adenocarcinoma predominantly showing lepidic or acinar growth pattern. The High Risk Group included invasive mucinous adenocarcinoma, and invasive adenocarcinoma predominantly showing papillary or solid growth pattern.

Lung adenocarcinoma patients with different pathological subtypes were grouped into a High Risk Group and a Low Risk Group as previously described. On the basis of a Kaplan-Meier analysis, the median RFS was 22.5 months in the High Risk Group and not reached in the Low Risk Group (HR, 3.53; 95% CI, 1.24 to 10.08; *P* = 0.0059; **Figure 4A**). The median OS was 41.6 months in the High Risk Group and not reached in the Low Risk Group (HR, 6.36; 95% CI, 1.74 to 23.23; *P* = 0.0016; **Figure 4B**).

### Prognostic Analysis With Combination of Adenocarcinoma Subtypes and FR^+^CTC Levels

To assess the combinational effect of adenocarcinoma subtypes (Low vs. High Risk as previously defined) and FR^+^CTC levels on prognosis, lung adenocarcinoma patients were divided into four groups, Low Risk/Low FR^+^CTC level (Group 1, n = 8), Low Risk/High FR^+^CTC level (Group 2, n = 15), High Risk/Low FR^+^CTC level (Group 3, n = 2), and High Risk/High FR^+^CTC level (Group 4, n = 11). Group 3 was not included in the Kaplan–Meier analysis due to limited number of patients.

**Figure 5:**
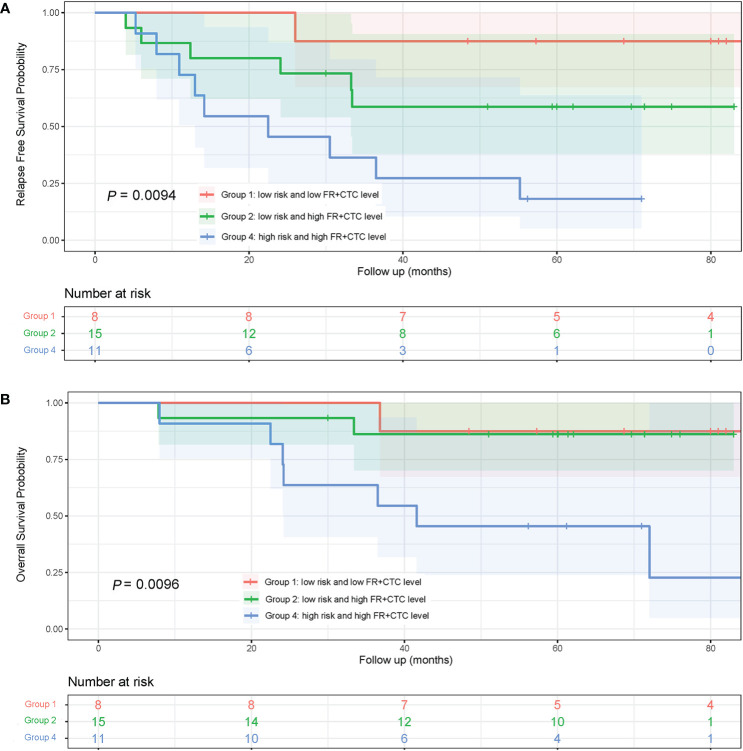
Kaplan-Meier curves of RFS and OS in lung adenocarcinoma patients with different subtypes and FR+CTC level combination. **(A)** RFS curves of patients with different risk of adenocarcinoma subtypes and FR^+^CTC level. **(B)** patients with different risk of adenocarcinoma subtypes and FR^+^CTC level.

The 5-year RFS rates of patients in Group 1, 2, and 4 were 87.5, 58.7, and 18.2%, respectively. The median RFS in Group 4 was significantly shorter than that in Group 1 (22.5 vs. >84 months; HR = 10.75; 95% CI, 3.10 to 37.31; *P* = 0.0043, **Figure 5A**) and that in Group 2 (22.5 vs. 28.0 months; HR = 2.62; 95% CI, 0.92 to 7.50; *P* = 0.056, **Figure 5A**). Since most patients in Groups 1 and Group 2 were still alive at the end of the seven-year follow up, the OS of the three groups were not compared (**Figure 5B**).

## Discussion

Surgical resection is one of the most effective treatment options for NSCLC, especially for early stage tumors. Outcomes from surgery are usually satisfactory, with 5-year survival rate ranging from 73–100% for Stage 0/I and 12–65% for Stage II/III cancer (3). Nonetheless, up to 50% of patients eventually develop recurrence within two years. When recurrence occurs, the 5-year survival rate drops dramatically to approximately 15% (3). As shown in this study, of the 21 patients in whom recurrence or metastasis developed, the median time from recurrence or metastasis to death was only 1.9 months. Therefore, it is critical to identify patients who are more susceptible to recurrence and proactively manage those patients during follow up in order to improve their outcomes. Currently, however, there is a lack of biomarkers that can effectively predict recurrence or metastasis such that healthcare providers can provide prompt and adequate treatments (26).

TNM staging is one of the most commonly used predictors for lung cancer and its clinical utilities have been proven repeatedly. However, TNM staging has its limitations. For example, in TNM staging, only tumor size, the degree of tumor invasion, and the extent of lymph node metastasis are considered, while other important tumor characteristics are not included (3). Circulating biomarkers, by providing additional aspects of the tumor, can supplement traditional pathological findings and help better understand the biological nature and the clinical implications of the tumor. Serological biomarkers such as carcinoembryonic antigen (CEA) and cytokeratin 19 soluble fragment (CYFRA21-1) are tumor associated antigens (TAAs) commonly used for NSCLC, but their utilities in early stage tumors and prognosis of surgical outcome are limited (27). CTC and ctDNA, on the other hand, have recently emerged as more promising circulating tumor biomarkers due to their non-invasiveness and direct biological relevance to the tumor. Stratifying patients using these new biomarkers is not only more convenient, but also can be more effective (28). A meta-analysis by Liang, et al. suggested that both post-operative CTCs and ctDNA were promising predictive biomarkers of tumor recurrence in NSCLC patients (29). Of these two, CTCs are more likely to be detected in early stage cancers due to low abundance and quick turnover of ctDNA in systemic circulation (28).

During surgical resection, while the primary tumor and mediastinal lymph nodes have been resected and no distant metastases have been observed, it is still possible that visually undetectable micro-metastases exist, which can later develop into recurrence or metastasis (30). Circulating tumor cells are tumor cells that are shed from the primary tumor and enter the circulation (31). CTCs are thought to play an important role in disease progression and metastasis (31). The number of CTCs and the proportion of which that can escape immune surveillance, relocate, seed, and proliferate at a different location of the body likely affect their ability to induce disease progression and metastasis (32, 33). For these reasons, CTC may be a potentially potent prognostic factor of disease recurrence and/or metastasis after lung cancer surgery.

The association between CTC and surgical outcome of resectable NSCLC patients has been investigated using different CTC detection technologies. In several studies, the authors suggested that postoperative CTC was an independent prognostic factor of DFS, but preoperative CTC was not (13, 34–36). Murlidhar et al. studied 36 patients with resectable NSCLC (stages I–III) and found that presence of clusters in preoperative peripheral blood predicted a trend toward poorer prognosis (37). Li et al. suggested that the number of CTCs in either peripheral blood or pulmonary vein blood was an independent risk factor for tumor free survival (TFS) and OS in NSCLC patients receiving surgical resection (38). Crosbie et al. found that CTC enumeration in pulmonary vein and peripheral blood combined during the operation better identifies NSCLC patients with a higher risk of recurrence than in peripheral blood alone (12). Chem et al. demonstrated that early-disseminating pulmonary venous CTCs were responsible for disease relapse (39).

For early stage lung cancer, sampling pulmonary vein blood for CTC analysis has been considered a better strategy due to the scarcity of CTCs and the suboptimal sensitivity of existing CTC detection technologies. However, intraoperative pulmonary vein blood collection is much more complicated than peripheral blood collection. Further, peripheral blood can be conveniently and repeatedly collected during a patient’s follow-up. Thus, peripheral blood would be the preferred source for CTC isolation and identification in clinical practice.

The negative enrichment and LT-PCR based FR^+^CTC detection has proven a sensitive method for CTC analysis in peripheral blood, with sensitivity of 70–90% for different stages of NSCLC (10, 11, 17, 40). Previous studies have also demonstrated that the FR^+^CTC is associated with tumor invasiveness and risk of recurrence for resectable NSCLC patients. In a multi-center study involving 382 patients, Zhou et al. found that the preoperative FR^+^CTC level was significantly lower in AIS and MIA than that in invasive pulmonary adenocarcinoma, the latter is known to have worse prognosis (20). In another study assessing different surgical procedures for early stage NSCLC patients, Wei et al. demonstrated that the vein-first ligation technique led to a significantly higher decrease in FR^+^CTC level after surgery than the artery-first ligation technique (41). Further, Wei et al. compared the long-term outcome of the two surgical procedures retrospectively and found that the former had better 5-year OS, DFS, and lung cancer specific survival (41). While these studies implicate that the FR^+^CTC level and its change pre- and post-surgery may be prognostic of patient outcome after surgical resection, such evidence is not direct as the studies lacked long-term outcome from the patients whose FR^+^CTC levels were measured.

To our knowledge, our study was the first one to assess the prognostic value of FR^+^CTC in long-term surgical outcome of resectable NSCLC patients. We demonstrated that preoperative FR^+^CTCs >7.9 FU/3 ml was an independent prognostic factor of DFS after R0 resection of NSCLC patients with a 7-year follow-up. High levels of preoperative FR^+^CTC were also associated with a numerically shorter OS although the association was not statistically significant.

The five-year survival rates of the postoperative patients with AIS, MIA, and lepidic predominant adenocarcinoma were approximately 100, 100, and 90%, respectively, while those of other subtypes of adenocarcinoma including mucinous, micropapillary, and solid subtype were significantly lower (42). In this study, we observed a similar trend. Further, when patients were classified into high- and low-risk groups based on these pathological subtypes, a significant higher FR^+^CTC level was seen in the high-risk group (including mucinous, micropapillary, and solid). Such a finding was not surprising as the pathological subtypes reflect tumor invasiveness or proliferation characteristics and CTC level also reflects active tumor burden. Interestingly, when both FR^+^CTC level and adenocarcinoma subtypes were assessed, both factors were significantly associated with long-term surgical outcome. Furthermore, patients with both high FR^+^CTC level and high risk pathological subtypes had a nine-fold increase in risk of disease recurrence than those with both low FR^+^CTC level and low risk pathological subtypes. These results suggest that FR^+^CTC provides additional insights in recurrence risk beyond those from conventional pathological evaluation.

The present study has several limitations. First, the study was conducted in a single center and has limited sample size in part due to difficulties in patient enrollment at the initiation of the study and censoring during the 7-year follow-up. As such, the results from the study requires further validation in larger, multi-center investigations. Further, all patients received FR+CTC analysis prior to surgery but lacked longitudinal assessment of FR+CTC levels post-treatment, either during postoperative hospital stay or subsequent follow-ups. Therefore, the prognostic effect of the dynamic change in FR+CTC level cannot be assessed. Nor can the association between the degree or timing of FR+CTC change and tumor recurrence or progression be evaluated. These questions are of particular interest to thoracic surgeons as they provide direct insights on how to better manage NSCLC patients post-operation and should be addressed in future larger, multi-center studies.

In conclusion, our study demonstrated that preoperative FR^+^CTC level was a potential predictor for the prognosis in NSCLC patients underwent surgery. Interestingly, while baseline FR^+^CTC level was associated with primary tumor invasion or proliferation characteristics, its prognostic value appears to go beyond those well-known pathological features and thus warrants further investigation in larger, systematic studies.

## Data Availability Statement

The raw data supporting the conclusions of this article will be made available by the authors, without undue reservation.

## Ethics Statement

The studies involving human participants were reviewed and approved by Shanghai Cancer Center Fudan University (050432-4-1911D). The patients/participants provided their written informed consent to participate in this study. Written informed consent was obtained from the individual(s) for the publication of any potentially identifiable images or data included in this article.

## Author Contributions

HL, WH, and HH made substantial contributions to the conception or design of the work. YawZ, YS, and XY collected and detected the samples. HL, BL, YanZ, YP, and JX acquired the data. HL, BL, YanZ, and WH analyzed or interpreted the data. HL, BL, XY, and HH drafted the work or revised it critically for important intellectual content. All authors contributed to the article and approved the submitted version.

## Funding

This work was supported by the National Natural Science Foundation of China (82003285) and Shanghai Anticancer Association EYAS project (SACA-CY1A03).

## Conflict of Interest

XY and WH were employed by the company Geno Biotech Co. Ltd.

The remaining authors declare that the research was conducted in the absence of any commercial or financial relationships that could be construed as a potential conflict of interest.

## Abbreviations

AIS, adenocarcinoma in situ; FR^+^CTC, folate receptor-positive circulating tumor cell; LT-PCR, ligand-targeted polymerase chain reaction; MIA, minimally invasive adenocarcinoma; NSCLC, non-small cell lung cancer; OS, overall survival; PCR, polymerase chain reaction; RFS, relapse-free survival; SD, standard deviation.
